# Is a second TUR necessary in patients with primary high‐grade Ta NMIBC, particularly in the context of initial cases?

**DOI:** 10.1002/bco2.70082

**Published:** 2025-09-14

**Authors:** Satoki Abe, Hiroyuki Fujinami, Naoyuki Yamanaka, Shinro Hata, Toru Inoue, Tadasuke Ando, Toshitaka Shin

**Affiliations:** ^1^ Department of Urology, Faculty of Medicine Oita University Yufu Oita Japan; ^2^ Organ Transplantation Promotion Project, Faculty of Medicine Oita University Yufu Oita Japan

**Keywords:** high‐grade Ta tumour, non‐muscle invasive bladder cancer, recurrence‐free survival, residual tumour rate, second transurethral resection

## Abstract

**Objective:**

To evaluate the clinical significance of a second transurethral resection of the bladder tumour (TURBT) in patients with a primary high‐grade (HG) Ta non‐muscle invasive bladder cancer (NMIBC), specifically selected for the initial diagnosis.

**Patients and Methods:**

We retrospectively analysed 121 patients with primary HG Ta urothelial carcinoma treated at our institution between January 2007 and October 2024. All patients underwent an initial TURBT with the detrusor muscle present in the specimen. Patients were divided into the second TUR group (n = 48) and the non‐second TUR group (n = 73). Propensity score matching was performed using age, number of tumours and Bacillus Calmette–Guerin treatment status. Outcomes included the residual tumour rate, recurrence‐free survival (RFS), time to progression to muscle invasive bladder cancer (MIBC) and cancer‐specific survival (CSS).

**Results:**

Residual tumour at the initial resection site was identified in four patients (8.3%) who underwent a second TUR, with two patients (4.2%) being upstaged to T1. The median follow‐up was 53 months. There were no significant differences between the two groups in RFS (p = 0.60), time to progression to MIBC (p = 0.63) or CSS (p = 0.18). These findings remained consistent in the matched cohort. Multivariate analysis revealed that a second TUR was not associated with improved RFS.

**Conclusions:**

This is the first study to specifically address primary HG Ta bladder cancer, and it suggests that a second TUR may be omitted in selected cases, particularly when the initial resection is complete and the detrusor muscle is adequately sampled. A risk‐adapted approach may help reduce unnecessary procedures without compromising oncological safety.

## 1 INTRODUCTION

Non‐muscle invasive bladder cancer (NMIBC) accounts for approximately 75% of all bladder cancer cases.^1^ Transurethral resection of the bladder tumour (TURBT) is the standard initial treatment for NMIBC. Among these, high‐grade (HG) Ta tumours are considered high risk due to their relatively high recurrence rates, and performing a second TUR is an option.

However, the necessity of a second TUR in HG Ta cases remains controversial. In T1 tumours, a second TUR is beneficial for detecting residual tumour, confirming accurate staging and improving prognosis.^2^, ^3^ In contrast, for HG Ta tumours with a complete initial resection, the clinical benefit of a second TUR is less clear. Several studies have reported a low residual tumour rate^4^ and minimal impact of a second TUR on oncological outcomes in these cases.^5^

In this study, we retrospectively evaluated the clinical outcomes of patients with primary HG Ta tumours and compared those who underwent a second TUR with those who did not. The objectives were to determine the residual tumour rate at the second TUR and to assess whether a second TUR contributes to recurrence or progression in this patient population.

## 2 PATIENTS AND METHODS

This retrospective, single‐centre cohort study was conducted between January 2007 and October 2024. The study was approved by the institutional review board (IRB No.2358). Patients who underwent TURBT for a first‐time diagnosis of bladder cancer and were pathologically confirmed to have primary HG Ta urothelial carcinoma (UC) were included. All included cases had a grossly complete resection, and the detrusor muscle present in the TURBT specimen. All patients received a single intravesical instillation of Pirarubicin immediately after TURBT. Patients with a follow‐up duration of less than six months were excluded. Patients were divided into two groups: the second TUR group, who underwent a second TUR after the initial TURBT, and the non‐second TUR group, who did not undergo a second TUR. The decision to perform a second TUR was at the discretion of the attending physicians.

### 2.1 Outcome

The outcomes of this study included the residual tumour rate, recurrence‐free survival (RFS), time to progression to muscle invasive bladder cancer (MIBC) and cancer‐specific survival (CSS). The residual tumour rate was defined as the presence of any residual UC (excluding carcinoma in situ) detected at the site of the initial tumour resection during the second TUR. Pathological upstaging to T1 at the time of the second TUR was also recorded. RFS was defined as the time from the date of the primary TURBT for HG Ta tumours to the first confirmed recurrence of any UC during follow‐up. Time to progression to MIBC was defined as the time from the initial TURBT to the pathological diagnosis of stage ≥T2 disease. CSS was defined as the time from the initial TURBT to death attributable to UC, with patients who died from other causes censored at the time of death. Follow‐up surveillance was performed according to institutional protocols, typically consisting of cystoscopy and urine cytology every three months for the first two years and every six months thereafter, up to five years postoperatively.

### 2.2 Statistical analysis

Patient characteristics were compared between the two groups using Fisher's exact test for categorical variables, and the Mann–Whitney U test for continuous variables. To reduce selection bias, propensity score matching (PSM) was performed using variables such as age, sex, number of tumours and Bacillus Calmette–Guerin (BCG) treatment status. One‐to‐one nearest‐neighbour matching without replacement was applied. Survival curves for RFS, time to progression to MIBC and CSS were estimated using the Kaplan–Meier method and compared using the log‐rank test. A Cox proportional hazards model was used to identify independent predictors of recurrence. A two‐sided p‐value of <0.05 was considered statistically significant. All statistical analyses were performed using EZR software, version 1.68.

## 3 RESULTS

**FIGURE 1:**
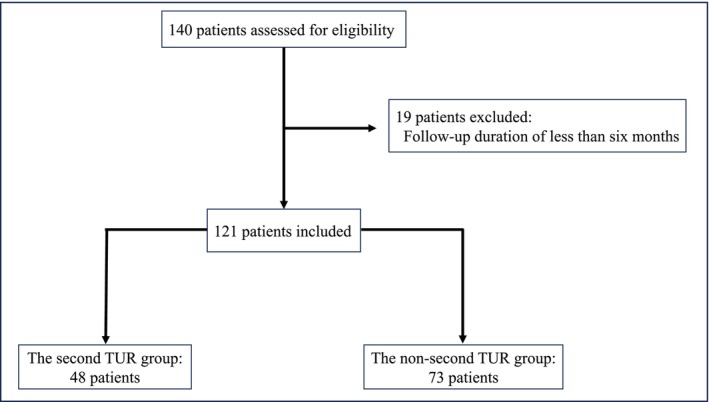
Flowchart of the study population.

**FIGURE 2:**
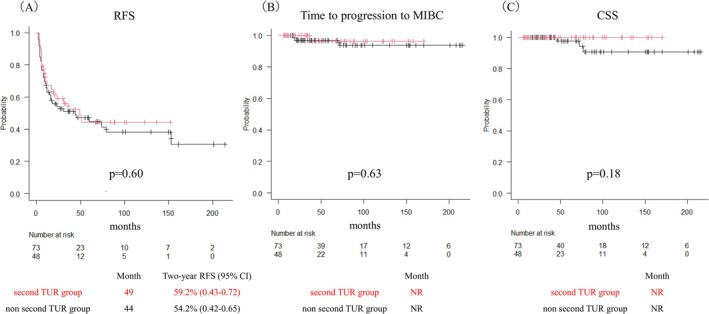
Kaplan–Meier curves before propensity score matching depicting (A) recurrence‐free survival (RFS), (B) time to progression to muscle invasive bladder cancer (MIBC) and (C) cancer‐specific survival (CSS) according to second TUR status.

**FIGURE 3:**
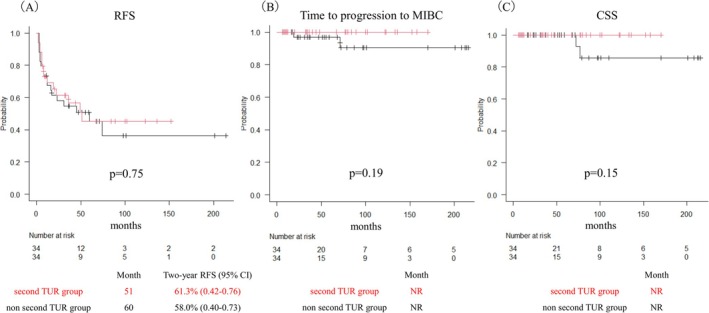
Kaplan–Meier curves after propensity score matching depicting (A) recurrence‐free survival (RFS), (B) time to progression to muscle invasive bladder cancer (MIBC) and (C) cancer‐specific survival (CSS) according to second TUR status.

A total of 121 patients with primary HG Ta UC were included in the analysis. Of these, 48 patients (39.7%) underwent a second TUR (the second TUR group), while 73 patients (60.3%) did not (the non‐second TUR group) (Figure 1). Baseline characteristics were generally balanced between the two groups, although differences were observed in age, tumour multiplicity and BCG treatment. PSM was performed using variables such as age, number of tumours and BCG treatment, resulting in two matched cohorts of 34 patients each (Table 1). Among the 48 patients who underwent a second TUR, residual tumour was identified in four patients (8.3%). Of these, two were upstaged to pT1. Progression to MIBC occurred in one patient (2.1%) in the second TUR group and in three patients (4.1%) in the non‐second TUR group. The median follow‐up duration was 53 months. The median RFS was 49 months in the second TUR group and 44 months in the non‐second TUR group (p = 0.60). The two‐year RFS rates were 59.2% and 54.2%, respectively. The median time to progression to MIBC was not reached in either group, and no significant difference was observed (p = 0.63). CSS was also not reached, with no significant difference between the groups (p = 0.18) (Figure 2). In the matched cohort, the median RFS was 51 months in the second TUR group and 60 months in the non‐second TUR group (p = 0.75). There were no significant differences in time to progression to MIBC (p = 0.19) or CSS (p = 0.15) (Figure 3). Multivariate Cox regression analysis demonstrated that a second TUR was not significantly associated with improved RFS (hazard ratio: 1.13; 95% confidence interval [CI]: 0.62–2.05; p = 0.70) (Table 2). Other variables, including tumour multiplicity and BCG treatment, were also not identified as independent predictors of recurrence.

**TABLE 1:** Characteristics of patients with high‐grade Ta bladder cancer at initial TURBT.

Characteristics	Before propensity score match			After propensity score match		
	Second TUR group (*N* = 48)	Non‐second TUR group (*N* = 73)	*p*‐value	Second TUR group (*N* = 34)	Non‐second TUR group (*N* = 34)	*p*‐value
Age (years), median (range)	71 (51–83)	76 (51–94)	0.01	71 (51–83)	72 (51–86)	0.54
Sex, *N* (%)						1
Male	44 (91.7)	58 (79.5)	0.08	31 (91.2)	31 (91.2)	
Female	4 (8.3)	15 (20.5)		3 (8.8)	3 (8.8)	
eGFR (mL/min/1.73 m²), median (range)	57.8 (11.0–97.9)	59.9 (4.9–101.7)	0.38	65.5 (11.0–97.9)	60.9 (13.8–101.7)	0.60
Number of tumours, *N* (%)						047
1	15 (31.3)	41 (56.2)	0.009	15 (44.1)	19 (55.9)	
≧2	33 (68.7)	32 (43.8)		19 (55.9)	15 (44.1)	
Past UTUC, *N* (%)	6 (12.5)	15 (20.5)	0.33	4 (11.8)	1 (2.9)	0.36
Urine cytology, *N* (%)						1
≦Class 3a	11 (22.9)	27 (37.0)	0.11	8 (23.5)	8 (23.5)	
≧Class 3b	37 (77.1)	46 (63.0)		26 (76.5)	26 (76.5)	
PDD (5‐ALA), *N* (%)	3 (6.3)	3 (4.1)	0.68	2 (5.9)	1 (2.9)	1
CIS concomitant, *N* (%)	14 (29.2)	13 (17.8)	0.18	9 (26.5)	12 (35.3)	0.60
BCG induction treatment, *N* (%)	23 (47.9)	12 (16.4)	<0.001	9 (26.5)	11 (32.4)	0.79
BCG maintenance treatment, *N* (%)	5 (10.4)	3 (4.1)	0.26	3 (8.8)	3 (8.8)	1

5‐ALA, 5‐amino levulinic acid; BCG, Bacillus Calmette–Guerin; PDD, photodynamic diagnosis; UTUC, upper tract urothelial carcinoma.

**TABLE 2:** Univariable and multivariable Cox regression analyses of clinical and treatment‐related variables associated with recurrence‐free survival before propensity score matching.

		Univariate analysis		Multivariate analysis	
		HR (95% CI)	*p*‐value	HR (95% CI)	*p*‐value
Age (year)	<75 vs. ≧75	1.70 (1.02–2.84)	0.04	1.59 (0.91–2.77)	0.10
eGFR (mL/min/1.73 m²)	≧60 vs. <60	1.16 (0.71–1.91)	0.55	1.01 (0.59–1.74)	0.96
Urine cytology	≦Class3a vs. ≧Class3b	1.12 (0.66–1.92)	0.67	1.37 (0.78–2.40)	0.27
Number of tumours	1 vs. ≧2	1.09 (0.66–1.79)	0.75	1.20 (0.71–2.05)	0.49
Past UTUC	Yes vs. no	1.34 (0.71–2.52)	0.37	1.32 (0.66–2.62)	0.43
CIS concomitant	Yes vs. no	0.32 (0.14–0.73)	0.007	0.43 (0.15–1.24)	0.12
Second TUR	Yes vs. no	0.87 (0.52–1.47)	0.61	1.13 (0.62–2.05)	0.70
BCG induction therapy	Yes vs. no	0.41 (0.21–0.79)	0.007	0.59 (0.23–1.48)	0.26
BCG maintenance therapy	Yes vs. no	0.38 (0.09–1.56)	0.18	0.81 (0.17–3.79)	0.79

BCG, Bacillus Calmette–Guerin; CI, confidence interval; HR, hazard ratio; UTUC, upper tract urothelial carcinoma.

## 4 DISCUSSION

Current international guidelines recommend a second TUR for all HG NMIBC, largely based on data from T1 tumours, due to the high likelihood of residual disease and the potential for understaging in these cases. However, there is growing debate as to whether these recommendations are applicable to HG Ta tumours. The European Association of Urology and the French Association of Urology guidelines do not recommend a second TUR for HG Ta tumours when the initial resection includes the detrusor muscle.^6^, ^7^ In contrast, the American Urological Association recommends a second TUR in the same setting, at least for larger and multifocal tumours.^8^ The Japanese guidelines also recommend considering a second TUR for HG Ta tumours.^9^ The main reason for the conflicting recommendations regarding the second TUR for HG Ta UC across guidelines is likely related to differences in the inclusion criteria, particularly with respect to the quality of the initial TURBT.

Lee K. et al.^10^ reported a higher two‐year RFS rate (81.3% vs. 60.1%, p = 0.005) in a cohort of 187 patients who underwent macroscopically complete initial TURBT. However, in that study, the presence of detrusor muscle in the initial TURBT was not reported, which may represent a potential explanation for the higher recurrence risk observed in the single TURBT group. Hensley et al.^11^ reported improved RFS (not reached vs. 64 months [37–106], p = 0.003) in favour of a second TUR in a cohort of 209 patients who received adequate adjuvant BCG therapy. However, patients without detrusor muscle in the initial TURBT specimen were not excluded, which may indicate that the effect of a second TUR on recurrence and progression has been overestimated.

Mebroukine S et al. established very strict selection criteria, excluding patients with a history of previous HG Ta or T1 tumour, incomplete initial TURBT or absence of detrusor muscle in the first resection specimen.^5^ Similarly, in our study, by including only patients with detrusor muscle present in the initial TURBT specimen, we were able to evaluate the true clinical significance of a second TUR. Moreover, previous studies did not exclude patients with a history of low‐grade UC, nor did they clarify whether such a history was present. To our knowledge, this is the first study to strictly include only patients with primary HG Ta NMIBC, with a specific focus on initial cases.

In this retrospective study, we evaluated the clinical significance of a second TUR in patients with primary HG Ta NMIBC. Our results demonstrated that the residual tumour rate at the site of the initial resection was low (8.3%), and pathological upstaging to T1 occurred in only two patients (4.2%). Previous reports have shown a residual tumour rate of 17–67% on second TUR for HG Ta UC.^2^, ^4^, ^5^, ^11^ In contrast, our study demonstrated a substantially lower residual rate. In some previous studies, residual tumours included cases of CIS; however, even after excluding CIS, the residual rate in our cohort remained low. This may be attributable to the fact that complete resection and inclusion of the detrusor muscle were consistently achieved during the initial TURBT.

Furthermore, there were no statistically significant differences in RFS, time to progression to MIBC or CSS between the second TUR and the non‐second TUR groups, even after PSM. Our findings support a more selective approach, suggesting that routine second TUR may not be necessary for all HG Ta cases, particularly when the initial resection is complete and detrusor muscle is present in the specimen.

In this study, performing a second TUR for HG Ta tumours did not contribute to the prevention of recurrence. Mebroukine S et al. have suggested that hexaminolevulinate‐based photodynamic diagnosis (HAL‐PDD) TURBT and adjuvant therapies such as BCG or Mitomycin C (MMC) are effective in reducing recurrence.^5^ In our study, although the recurrence rate was relatively high, the implementation rate of PDD TURBT was low. Therefore, increasing the use of PDD TURBT in the future may contribute to improved recurrence control.

A phase III trial comparing transurethral en bloc resection of the bladder tumour (ERBT) with conventional TURBT reported that a second TUR could be avoided if resection margins were negative in ERBT.^12^ If ERBT replaces conventional TURBT as the standard procedure for NMIBC in the future, a second TUR may no longer be necessary for HG Ta tumours with negative resection margins.

This study has several limitations. First, it was a single‐centre, retrospective study with inherent selection bias, although we attempted to mitigate this by using PSM. Second, the sample size was relatively small, particularly in the matched cohort, which may have limited the statistical power to detect small but clinically meaningful differences in outcomes. A prospective multicentre study or a randomised controlled trial is warranted to validate these findings. Third, the timing of the second TUR was not standardised, with some procedures performed beyond the typical 2–6‐week window. Lastly, we included only primary HG Ta cases, and therefore, our findings may not be generalisable to patients with recurrent tumours.

Despite these limitations, our results suggest that a second TUR may not be essential for all patients with primary HG Ta tumours, and individualised decision‐making based on tumour characteristics and the quality of the initial resection should be considered.

## 5 CONCLUSIONS

In patients with primary HG Ta tumours, the rate of residual tumour at second TUR was low, and a second TUR did not significantly impact RFS, time to progression to MIBC or CSS. These findings suggest that routine second TUR may be omitted in selected patients who have undergone complete initial resection with adequate sampling of the detrusor muscle. A risk‐adapted approach should be considered to reduce unnecessary procedures while maintaining oncological safety.

## AUTHOR CONTRIBUTIONS

Satoki Abe contributed to study design, data collection, data analysis and drafting of the manuscript. Hiroyuki Fujinami, Naoyuki Yamanaka, Shinro Hata, Toru Inoue and Tadasuke Ando contributed to the review of the manuscript. Toshitaka Shin contributed to supervision of the study and critical revision of the manuscript. All authors read and approved the final version of the manuscript.

## CONFLICT OF INTEREST STATEMENT

The authors declare no conflicts of interest.
